# Prevalence and correlates of depression among the trans-genders of Pakistan

**DOI:** 10.1192/j.eurpsy.2021.1594

**Published:** 2021-08-13

**Authors:** U. Zubair

**Affiliations:** Oak, phoenix care center, Dublin, Ireland

**Keywords:** depression, socio-demographic factors, trans-genders

## Abstract

**Introduction:**

Census conducted by government of Pakistan in 2017 has shown that more than 10000 trans-genders live in Pakistan. HIV, illicit substance use and mental health issues including depression are the main health problems faced by this part of coummunity

**Objectives:**

To assess the prevalence of depression among the transgender population and analyze the relationship of socio-demographic factors with depression.

**Methods:**

The sample population comprised of one hundred and forty two transgender people of Rawalpindi and Islamabad. Beck depressive inventory II (BDI-II) was used to record the presence and severity of the depressive symptoms. Depressive symptoms were categorized as mild, moderate and severe. Relationship of the age, smoking, family income, illicit substance use and education was studied with the presence of depressive symptoms among these transgender population of twin cities of Pakistan

**Results:**

A total of 142 transgender people were included in the final analysis. Mean age of the study participants was 39.55 ± 6.18. Out of these, 45.1% had no depressive symptoms while 31.7% had mild, 12.7% had moderate and 10.6% had severe depressive symptomatology. After applying the binary logistic regression we found that presence of depressive symptoms had significant association with illicit substance use among the target population.

**Conclusions:**

This study showed a high prevalence of depressive symptoms among transgender population of twin cities of Pakistan. Use of illicit substances like tobacco, cannabis, opiates and alcohol should be discouraged and those using these should be routinely screened for the presence of other mental health issues in order to timely diagnose and treat them

